# Species identification and drug susceptibility testing of non-tuberculous mycobacteria by Line Probe Assay in Lambaréné, Gabon—a cross-sectional study

**DOI:** 10.1186/s12879-023-08617-x

**Published:** 2023-10-03

**Authors:** Micheska Epola Dibamba Ndanga, Jabar Babatundé Pacome Achimi Agbo Abdul, Jean Ronald Edoa, Guy Arnault Rogue Mfoumbi Ibinda, Bayodé Romeo Adegbite, Rhett Chester Mevyann, Christopher Mebiame Biyogho, Jocelyn Mahoumbou, Stredice Manguinga, Nina Mbenga Roguet, Bertrand Lell, Peter Gottfried Kremsner, Abraham Sunday Alabi, Martin Peter Grobusch, Ayola Akim Adegnika

**Affiliations:** 1 Centre de Recherches Médicales de Lambaréné (CERMEL), Lambaréné, Gabon; 2 Ecole Doctorale d’infectiologie Tropicale de Franceville, Franceville, Gabon; 3 Center of Tropical Medicine and Travel Medicine, Department of Infectious Diseases, Amsterdam University Medical Centers, Location University of Amsterdam, Amsterdam Infection & Immunity, Amsterdam Public Health, University of Amsterdam, Amsterdam, The Netherlands; 4 Programme National de Lutte Contre La Tuberculose, Libreville, Gabon; 5 Department of Medicine I, Division of Infectious Diseases and Tropical Medicine, Medical University of Vienna, Vienna, Austria; 6 German Center for Infection Research, Tübingen, Germany; 7 Institut Für Tropenmedizin, Universität Tübingen, Tübingen, Germany; 8 Health Focus GmbH, Potsdam, Germany; 9 Masanga Medical Research Unit (MMRU), Masanga, Sierra Leone; 10 Institute of Infectious Diseases and Molecular Medicine, University of Cape Town, Cape Town, South Africa; 11 Fondation Pour La Recherche Scientifique, Cotonou, Bénin; 12 Department of Parasitology, Leiden University Medical Center, Leiden, The Netherlands

**Keywords:** Non-tuberculous mycobacteria (NTM), Genotypic drug susceptibility testing, Genotypic NTM-DR, Macrolides, Aminoglycosides, Gabon

## Abstract

**Background:**

Non-tuberculous mycobacteria (NTM) are a group of bacteria that cause rare lung infections and are increasingly recognized as causative agents of opportunistic and device-associated infections in humans. In Gabon, there is a lack of data on NTM species identification and drug susceptibility. The aim of this study was to identify the frequency of NTM species and their genotypic susceptibility pattern to commonly used antibiotics for NTM infections in Gabon.

**Methods:**

A cross-sectional study was conducted at the CERMEL TB laboratory from January 2020 to December 2022, NTM subspecies identification and drug susceptibility testing to macrolides and aminoglycosides were performed using the genotype NTM-DR kit.

**Results:**

The study found that out of 524 culture-positive specimens, 146 (28%) were NTM, with the predominant group being *Mycobacterium avium* complex (MAC) and *Mycobacterium abscessus* complex (MABC). All MAC isolates were fully susceptible to macrolides and aminoglycosides, while five MABC isolates carried mutations indicative of reduced susceptibility to macrolide and aminoglycoside drugs.

**Conclusions:**

These findings suggest that clinicians may use macrolides and aminoglycosides to manage NTM infections caused by MAC, but further investigation is required to determine MABC drug susceptibility.

## Background

Non-tuberculous mycobacteria (NTM) are mycobacteria other than *M. tuberculosis* and *M. leprae*. They are environmental pathogens predominantly found in water, soil, dust and animal sources [1]. In the past, NTM infections did not attract much attention since they did not exhibit human-to-human transmission. Very often, isolates were considered as contaminants by laboratories, and were therefore overlooked [2, 3].

Moreover, their epidemiology has been difficult to establish because reporting of NTM infections was not mandatory in most parts of the world [4]. Advanced diagnostic laboratory techniques, as well as increased clinicians’ awareness certainly contribute to a recent increase in worldwide reporting of their incidence, which is estimated to be between 1.0 and 1.8 per 100,000 people per year [5]. However, one determining factor behind the evolution of the epidemiology of NTMs is the emergence of the human immunodeficiency virus. In fact, people living with HIV, cystic fibrosis (CF), bronchiectasis, emphysema and chronic obstructive pulmonary disease (COPD) constitute the high-risk population for NTM infections [3, 5–7].

There are more than 170 NTM species identified to date, but only a few species are believed to be opportunistic pathogens and responsible for human infections. The most-common of these species are *M. avium complex* (MAC*), M. abscessus* complex (MABC), *M. kansasii*, *M. fortuitum*, *M. chelonae, M. szulgai, M. triviale* and *M. scrofulaceum*.

Infections caused by NTM are diverse and present in both immunocompromised and immunocompetent patients. Bone infections, skin infections and disseminated disease have been described; 90% of which are chronic lung infections in the same way as tuberculosis [8]. In fact, clinically, it is difficult to discern the symptoms of NTM lung disease and tuberculosis. This can lead to incorrect diagnoses in the absence of microbiological identification. Moreover, NTM are resistant to several antimycobacterial agents; hence the low efficacy of the latter [9, 10].

In order to inform clinical decision-making to optimise treatment efficacy, as the intrinsic antibiotic resistance profile is species-specific [5, 11], species identification must precede sensitivity profile determination. Macrolides and aminoglycosides have been considered first-choice molecules in the management of NTM infection according to the American Thoracic Society/Infectious Diseases Society of America (ATS/IDSA) [5].

In high-income countries, the management of NTM can be considered routine [5]. However, in the sub-Saharan region, there is very little data on NTM management and drug susceptibility profiles, as very few laboratories perform these tests. Most studies are focused on the prevalence of pulmonary NTM. In 2017, a systematic review and meta-analysis of 37 articles on NTM in the South-of the-Sahara region revealed a prevalence of 7.5% of pulmonary NTM, with MAC as the predominant species [12].

Like the other sub-Saharan countries, Gabon has some data on the isolation of NTM. Indeed, a study conducted by our group in 2022 showed a proportion of 10% of NTM in patients presumed to have tuberculosis in the Lambaréné region, with MAC being the most-common species [13]. Whilst antimicrobial susceptibility information is essential for clinicians to select appropriate treatment regimens, drug susceptibility testing for NTM species has not been widely conducted in Gabon.

Thus, the objective of this study was to identify the species and drug susceptibility profiles of NTM commonly isolated at the Tuberculosis Reference Laboratory of the Lambaréné Medical Research Center (CERMEL).

## Methods

### Study site

This study was carried out at the CERMEL TB laboratory, which is the national TB reference laboratory of Gabon. The laboratory receives samples from laboratories in peripheral areas for routine surveillance of drug-resistant tuberculosis. In addition, the laboratory performs susceptibility testing (Line Probe Assay and conventional DST) and monitors MDR-TB patients from the whole country using a culture technique [14–16].

### Study design

This was a prospective cross-sectional study of isolates from positive cultures of presumptive TB patients identified as NTM species, from January 2020 to December 2022. A limited number of specimens collected in 2020 in the framework of an earlier-reported study were also included in this analysis [13].

### Sampling

Two sputum samples of each presumptive TB patient were collected over two consecutive days. Specimens that were positive after culture were specified as NTM using Genotype CM/AS version 2.0 (Bruker Hain Lifescience, Nehren, Germany). Only specimen from patients with species found to be identical in each of the culture isolates were eligible for susceptibility testing.

### Data collection

All data were collected from patients using a structured questionnaire and on the Laboratory test request form. Demographic data (age, sex), clinical information (HIV status, history of TB treatment, clinical symptoms), AFB smear results, and radiological results were collected. Study data were managed using REDCap (Research Electronic Data Capture) [17, 18].

### Extraction and species identification

DNA was extracted from a positive mycobacterial culture using the Genolyse kit (Hain Lifescience, Nehren, Germany). The GenoType CM/AS kit (Hain Lifescience, Nehren, Germany) was used to prepare the master mix for polymerase chain reaction (PCR) amplification of the speciation-determining region using biotinylated primers present in the kit. After amplification, the labelled PCR products are hybridised with specific oligonucleotide probes immobilised on a strip. The post-hybridisation reaction results in the appearance of coloured bands on the band at the probe binding site and can be observed with the naked eye. Labelled hybrids captured and detected by colorimetric development can be used to detect different species. All the assays were performed according to the manufacturer's instructions [19].

### Genotypic drug susceptibility testing of NTM (GenoType NTM-DR test)

In vitro antimicrobial susceptibility by the broth microdilution testing of NTM was not performed within the realm of this study. We performed the genotypic method with the GenoType NTM-DR assay, a qualitative in vitro test assay based on PCR and DNA strip technology. Mycobacterial DNA is extracted from cultivated material, specifically amplified via PCR, and detected on membrane strips using reverse hybridisation and an enzymatic colour reaction. The Genotype NTM-DR assay permits the simultaneous genetic detection of several relevant NTM: *M. avium, M intracellulare, M chimaera, M. chelonae and the M. abscessus* complex (*M. abscessus* subsp. *abscessus, M. abscessus* subsp. *massilliense and M. abscessus* subsp. *bolletii*); their resistance to aminoglycosides (kanamycin, amikacin, gentamicin) via the detection of the most relevant mutations of the *rrs* gene; and their resistance to macrolides (clarithromycin, azithromycin) via the detection of the most relevant mutations of the *rrl* gene. Additionally, the *erm*(41) gene is analysed for the identification of macrolide resistance in members of the *M. abscessus* complex. The gene is divided into two probes; the *erm*(41*)* C28 probe and the *erm*(41) T28 probe. When the *erm*(41) C28 probe is positive, this indicates that the strain tested is susceptible to macrolides (except for strains with the additional *rrl* mutation). When the *erm*(41) T28 probe is positive, this indicates that the strain tested is resistant to macrolides, and the *rrl* gene is also examined for the detection of resistance to macrolides (clarithromycin or azithromycin). The *rrs* gene is examined for polymorphisms indicative of resistance to aminoglycosides (kanamycin, amikacin, gentamicin).

### Data analysis

Data analysis was performed using R version 4.0.1; the dplyr package was used for calculus methods. Chi-square tests were performed to establish a relationship between the categorical or binary variables. The factors associated with positive NTM status were quantified by logistic regression. The model building process followed a backward stepwise strategy whereby univariable analysis of all variables preceded the multivariable analysis. A variable was retained in the model if it was significant (*p* < 0.05). Two-sided *p*-values of 0.05 or less were considered statistically significant.

## Study results

### NTM species identification

The specimens in this study came from 524 culture positive samples among presumptive TB patients, including 29 fast-growing samples collected in 2020 from a previous study [13]. A total of 146 isolates (27.9%) were identified as NTM between February 2020 to November 2022. Of the 146 isolates confirmed as NTM, 142 (97.3%) were from sputum, while four (2.2%) were from urine samples. Four species (complexes) were identified among the isolates, and the predominant group was *M. avium complex* (MAC 80/146; 54.8%; of which *M. intracellulare* 53/146 (36.3%) and *M. avium* 27/146 (18. 5%)). *M. abscessus* complex (MABC) amounted to 38/146 (26.0%) of the isolates. However, in 11 cases (7.5%), there were two species detected. GenoType Mycobacterium NTM-DR could identify the subspecies of MABC *M. abscessus* subsp. *abscessus* (20 isolates), *M. abscessus* subsp. *bollettii* (8*),* and *M. abscessus* subsp*. massiliense* (10). The isolated species are listed in Table 1.

**Table 1:** Distribution of banding pattern and NTM species among 146 presumptive TB patients analysed

Band pattern CM/AS^a^	Band pattern NTM-DR^b^	Species	Frequency (%)
1,2,3,9	SP2	*M. intracellulare*	53 (36.3)
1,2,3,4	SP1	*M. avium*	27(18. 5)
1,2,3,5,10	SP4,SP5	*M. chelonae*	17 (11.8)
1,2,3,5,6,10	SP4,SP5,SP6,SP9,SP10	*M. abscessus.* subsp*. abscessus*	20 (13.6)
1,2,3,5,6,10	SP5,SP6,SP7,SP9,SP10	*M. abscessus.* subsp. *bolletii*	08 (05.4)
1,2,3,5,6,10	SP4,SP5,SP8,SP9,	*M. abscessus.* subsp. *massiliense*	10 (07.0)
1,2,3,4,9	NA	*M. intracellulare/M. avium*	03 (02.0)
1,2,3,5,6,9,10	NA	*M. abscessus/M. intracellulare*	03 (02.0)
1,2,3,4,5,6,10	NA	*M. intracellulare/M. avium*	03 (02.0)
1,2,3,5,6,7,9,10	NA	*M. abscessus/M. fortuitum*	02 (01.4)

*CM/AS* Common species /associate species, *SP* Specific probes, *NTM* Non tuberculous mycobacteria, *DR* Drug resistance, *NA* Not applicable, *TB* tuberculosis, *M. Mycobacterium*

^a^First NTM species identification

^b^Subspecies identification of MABC and MAC and drug resistance

### Patient characteristics

The average age of study participants was 46.8 (SD:14) years; 88/142 (62.0%) of the patients were male; 47/142 (33.1%) patients were HIV-co-infected. Regarding treatment history, 32/142 (22. 5%) of the NTM patients isolates had previously been treated for TB. Among the positive NTM isolates, only 34/142 (23.9%) of the smears were positive (Table 2). The clinical characteristics of NTM infections of male and female patients are compared in Table 3. The results show that the proportion of cough and hemoptysis in male patients were significantly higher than in female patients (93% versus 36%, *p* < 0.030; 37% vs 20%, *p* < 0.040, respectively).

**Table 2:** Characteristics of the 142 patients found positive to the NTM among presumptive TB patients

Characteristics	Number of patients
Gender	
Male	88 (62.0%)
Female	54 (38.0%)
Mean age (years)	46.8
Co-morbidities	
Diabetes	8 (5.6%)
HIV + status	47 (33.1%)
History of TB	32 (22. 5%)
Clinical presentation	
Productive cough	125 (88. 0%)
Haemoptysis	44 (31. 0%)
Laboratory examinations	
AFB smear positive	34 (23. 9%)
Radiological features	
Infiltrations	111 (78. 1%)
Cavities	134 (94. 3%)

*TB* tuberculosis, *HIV* human immunodeficiency virus, *AFB* acid-fast bacilli. MTB/RIF

**Table 3:** Comparison between male and female pulmonary NTM isolates patients among presumptive tuberculosis patients

Characteristics	Male (*N* = 88)	Female *N* = 54 (%)	*P* value
Co-morbidities			
History of TB	23 (26.1%)	9 (16.7)	0.219
HIV status	32 (36.4%)	15 (27.8)	0.359
Clinical presentations			
Productive Cough	82 (93.2%)	43 (36.4)	0.030
Haemoptysis	33 (37. 5%)	11 (20.4)	0.040
Laboratory investigations			
AFB smear	22 (25.0%)	12 (22. 2%)	0.840
Radiological features			
Cavities	84 (95.5%)	50 (37. 5%)	0.479
Infiltrations	67 (76.1%)	44 (81.5%)	0. 533

A difference between male and female patients with pulmonary NTM was considered statistically significant when *P* < 0.05

### Genotypic drug susceptibility testing of NTM species

Of the 146 NTM isolates, 135 were tested for antimicrobial susceptibility to macrolides (azithromycin, clarithromycin and capreomycin) and aminoglycosides (kanamycin, amikacin, and gentamycin) using GenoType NTM-DR. The other 11 species were not tested due to the multiple NTM species identified in the same isolate. Of the 38 isolates of the subspecies of the MABC complex, five isolates of the subspecies *M. abscessus* subsp. *abscessus* were found to be resistant to macrolides, with a mutation present in T28 in the *erm* probe (41), and a mutation in the rrl gene. Resistance to aminoglycosides was also found by a mutation in the rrs gene. The five strains that presented the *erm(41)* mutation also carried *rrl* and *rrs* mutations. However, no resistance-conferring mutations were detected for either MAC complex or *M. chelonae* (Table 4).

**Table 4:** Associated mutations and drug susceptibility results for macrolides and aminoglycosides in the GenoType1 Mycobacterium NTM-DR assay

Species/subspecies	Number of isolates	Gene						Macrolides	Aminoglycosides
		***erm* (41)**		***rrl***		***rrs***			
		**C28**	**T28**	**WT**	**MUT**	**WT**	**MUT**		
*M. intracellulare*	53	NA	NA	53	0	53	0	S	S
*M. avium*	27	NA	NA	27	0	27	0	S	S
*M. chelonae*	17	NA	NA	17	0	17	0	S	S
*M. abscessus* subsp. *massiliense*	10	10	0	10	0	10	0	S	S
*M. abscessus* subsp. *abscessus*	15	15	0	15	0	15	0	S	S
*M. abscessus* subsp. *abscessus*	5	0	5	5	5	5	5	R	R
*M. abscessus* subsp. *bolletii*	8	8	0	8	0	8	0	S	S

*erm* (41) the *erm* (41) gene is examined for detection of resistance to macrolides (clarithromycin or azithromycin) and is only present in members of the *M. abscessus* complex

*erm* (41) C28 probe detects a genotype that carries a C at position 28 of the *erm* (41) gene. When the *erm* (41) C28 probe stains positive, this indicates that the tested strain is sensitive to macrolides (except for strains with an additional rrl mutation)

*erm* (41) T28 probe detects a genotype that carries a T instead of a C at position 28 of the e*rm* (41) gene. When the *erm* (41) T28 probe stains positive, this indicates that the tested strain is resistant to macrolides

*rrl,* the *rrl* gene is examined for detection of resistance to macrolides (clarithromycin or azithromycin)

*WT* Wild type, *MUT* Mutation, *S* Susceptible, *R* Resistant and *NA* Not applicable

## Discussion

This study identified NTM species in presumptive tuberculosis patients in Gabon and determined their drug susceptibility profile using the LPA GenoType Mycobacterium NTM-DR test. The study revealed that *M. intracellulare* was the most-commonly occurring species, as observed previously in a study done in the same region, and also demonstrated in a study involving several countries in Sub-Saharan Africa [12, 13]. However, the MABC was the second most-common species isolated, which is contrary to the previous study where *M. fortuitum* was the second most-frequently found species. This describes the diversity of NTM species circulating among patients presumed to have tuberculosis in the Lambaréné region. As well, it has been documented that 25 to 60% of patients with a positive respiratory specimen for microbiological, respiratory signs and radiographic criteria have NTM pulmonary disease, and that due to its variable virulence, it is important to identify the species of NTM for better patient management [20].

In this study, results show that NTM isolates were more prevalent in male patients (88/142; 62%) as compared to female patients. This is in line with studies performed in Europe with 70%, and Tanzania 66%, which have suggested NTM pulmonary isolates to be originating mostly from men [21, 22]. However, some studies have indicated that pulmonary disease from NTM affects more women than men [23–25]. The findings from our study can be explained by the fact that our study population is based on cases of presumptive tuberculosis patients, and that in most studies on tuberculosis the male gender is the most-affected by the disease [22, 26]. Also, having a history of tuberculosis for pulmonary tuberculosis has been reported to be associated with NTM pulmonary isolates [13]. In this study, about 25% of the patients with NTM pulmonary isolates had a history of confirmed tuberculosis. Radiologically, the pulmonary presentation of NTM in our study was characterized by the presence of infiltrations and cavitations, which are also radiographic features similar to those of tuberculosis [27].

According to the Clinical Laboratory Standards Institute (CLSI) recommendations, drug susceptibility testing of NTM is usually done by the broth microdilution method as the gold standard. However, this method is time-consuming, given the delay due to the slow mycobacterial growth, as it is the case in *M. avium* [11]. In our study, 5/38 isolates of the MABC group were resistant to macrolides, with a mutation in the *erm*(41) gene at position T28 and mutation in the *rrl* gene in *M. abscessus* subsp. *abscessus*. These findings are similar to those of Maya et al*.,* which is due to the fact that *M. abscessus* subsp. *abscessus* and *M. abscessus* subsp. *bolettii* macrolide resistance is identified by polymorphisms at position 28 in the *erm*(41) gene (cytosine is replaced by thymine leading to resistance) and mutations at positions 2058/2059 (adenine is replaced by cytosine leading to resistance) in the *rrl* gene [22, 28, 29]. However, other species of this group such as *M. massiliense* have a non-functional *erm*(41) gene, do not have the target base at position 28 which results from a large deletion of a non-functional 276 bp, and therefore do not exhibit resistance to macrolides. To determine NTM macrolide resistance, it is necessary to check whether there is a mutation in the *rrl* gene [30, 31]. Five isolates of *M. abscessus* subsp. *abscessus were* resistant to aminoglycosides through the *rrs* gene. Management of MABC group NTM that are resistant to macrolides and aminoglycosides should be performed according to guidelines [32]. In our study, no mutation was detected in the MAC group (*M. avium, M. intracellulare*) indicating that MAC is sensitive to macrolides and aminoglycosides; in line with a study conducted in Ghana showing sensitivity of all MAC isolates to macrolides and aminoglycosides drugs [33].

This study has limitations. Firstly, the presence of two species of NTM in an isolate does not allow the genotypic susceptibility test to be carried out for those type of isolates, and the genotype can only be used for the species indicated in the kit, while the reference method (microdilution) can be used as a complement for the other species. Secondly, although macrolides and aminoglycosides are the first-line molecules for NTM lung disease, other molecules that can be combined with the therapeutic regimens have not been taken into account by the test used.

## Conclusions

Our study identified NTM species and tested drug susceptibility in routine culture isolates among tuberculosis patients in Lambaréné, Gabon. The study confirmed that the predominant NTM species was *M. intracellulare*, and that all MAC species were sensitive to macrolides and aminoglycosides. On the other hand, five MABC group isolates linked to the subspecies *M. abscessus* subsp. *abscessus* showed genotypical resistance to macrolides and aminoglycosides. Our research shows that NTM are present among presumptive TB cases in our setting, hence National Tuberculosis Control Programs should consider screening for NTM among presumptive TB cases. Knowledge of the NTM infection status and NTM drug susceptibility pattern is essential for correct diagnosis and selection of appropriate treatment regimens and should guide clinical decision-making.

## Data Availability

All data generated and analyzed during the current study are available from the corresponding author on reasonable request.

## Abbreviations

AFB: Acid Fast Bacilli
ATS: American Thoracic Society
CERMEL: Centre de Recherche Médicales de Lambaréné
CLSI: Clinical Laboratory Standards Institute DNA: deoxyribonucleic acid
DST: Drug Sensitivity Testing
HIV: Human Immunodeficiency Virus
IDSA: Infectious Diseases Society of America
LPA: Line Probe Assay
MABC: *Mycobacterium abscessus* Complex
MAC: *Mycobacterium avium* Complex
MDR-TB: Multidrug-resistant tuberculosis
NTM: Non-tuberculous mycobacteria
PCR: Polymerase chain reaction
REDCap: Research Electronic Data Capture
SD: Standard Deviation
TB: Tuberculosis
